# Angiotensin II type 1 receptor blocker telmisartan induces apoptosis and autophagy in adult T‐cell leukemia cells

**DOI:** 10.1002/2211-5463.12055

**Published:** 2016-04-13

**Authors:** Tomohiro Kozako, Shuhei Soeda, Makoto Yoshimitsu, Naomichi Arima, Ayako Kuroki, Shinya Hirata, Hiroaki Tanaka, Osamu Imakyure, Nanako Tone, Shin‐ichiro Honda, Shinji Soeda

**Affiliations:** ^1^ Department of BiochemistryFaculty of Pharmaceutical SciencesFukuoka UniversityJapan; ^2^ Department of Hematology and ImmunologyKagoshima University HospitalJapan; ^3^ Division of Hematology and ImmunologySchool of Medical and Dental SciencesCenter for Chronic Viral Diseases GraduateKagoshima UniversityJapan; ^4^ Department of Rheumatology and Clinical ImmunologyKumamoto University HospitalJapan; ^5^ Faculty of Sports and Health ScienceFukuoka UniversityJapan; ^6^ Department of Pharmaceutical Care and Health SciencesFaculty of Pharmaceutical SciencesFukuoka UniversityJapan

**Keywords:** adult T‐cell leukemia/lymphoma, apoptosis, autophagy, drug repositioning, human T‐cell leukemia virus‐1, telmisartan

## Abstract

Adult T‐cell leukemia/lymphoma (ATL), an aggressive T‐cell malignancy that develops after long‐term infection with human T‐cell leukemia virus (HTLV‐1), requires new treatments. Drug repositioning, reuse of a drug previously approved for the treatment of another condition to treat ATL, offers the possibility of reduced time and risk. Among clinically available angiotensin II receptor blockers, telmisartan is well known for its unique ability to activate peroxisome proliferator‐activated receptor‐γ, which plays various roles in lipid metabolism, cellular differentiation, and apoptosis. Here, telmisartan reduced cell viability and enhanced apoptotic cells via caspase activation in *ex vivo* peripheral blood monocytes from asymptomatic HTLV‐1 carriers (ACs) or via caspase‐independent cell death in acute‐type ATL, which has a poor prognosis. Telmisartan also induced significant growth inhibition and apoptosis in leukemia cell lines via caspase activation, whereas other angiotensin II receptor blockers did not induce cell death. Interestingly, telmisartan increased the LC3‐II‐enriched protein fraction, indicating autophagosome accumulation and autophagy. Thus, telmisartan simultaneously caused caspase activation and autophagy. A hypertension medication with antiproliferation effects on primary and leukemia cells is intriguing. Patients with an early diagnosis of ATL are generally monitored until the disease progresses; thus, suppression of progression from AC and indolent ATL to acute ATL is important. Our results suggest that telmisartan is highly effective against primary cells and leukemia cell lines in caspase‐dependent and ‐independent manners, and its clinical use may suppress acute transformation and improve prognosis of patients with this mortal disease. This is the first report demonstrating a cell growth‐inhibitory effect of telmisartan in fresh peripheral blood mononuclear cells from leukemia patients.

**Abbreviations**
ACs: asymptomatic HTLV‐1 carriers
AIF: apoptosis‐inducing factor
ARB: angiotensin II receptor blocker
ATL: adult T‐cell leukemia/lymphoma
CCR4: CC chemokine receptor 4
CICD: caspase‐independent cell death
CR: complete remission
HDs: healthy donors
HTLV‐1: human T‐cell leukemia virus
MOMP: mitochondrial outer membrane permeabilization
PBMCs: peripheral blood mononuclear cells
PPARγ: proliferator‐activated receptor‐γ
ROS: reactive oxygen species
WST‐8: water‐soluble tetrazolium‐8

Adult T‐cell leukemia/lymphoma (ATL), which has four categories (acute, lymphoma, chronic, and smoldering), is an aggressive peripheral T‐cell malignancy that develops after long‐term infection with human T‐cell leukemia virus (HTLV‐1) 1, 2, 3. HTLV‐1 infection has a worldwide distribution with endemic areas in Japan, Africa, Caribbean, Central and South America, where the majority of infected individuals remain asymptomatic carriers (ACs) and a minority develop hematologic or neurologic manifestations, i.e., ATL‐ or HTLV‐1‐associated myelopathy/tropical spastic paraparesis, respectively 1, 2, 4. In cases of acute, lymphoma, or unfavorable chronic subtypes (aggressive ATL), intensive chemotherapy like the LSG15 regimen is usually recommended 5. In spite of recent advances in chemotherapy, the prognosis for patients with ATL is one of the poorest among hematologic malignancies, even with allogeneic hematopoietic stem cell transplantation and supportive care; overall survival rate at 3 years is only 24% in more aggressive subtypes of ATL 6, 7, 8, 9. In cases of favorable chronic or smoldering ATL (indolent ATL), monitored waiting until the disease progresses has been recommended 5. Some patients with indolent ATL develop infections during this period. Therefore, an urgent need remains for therapy and prophylaxis of ATL 10, 11, 12, 13, 14.

In 2003, Ishida *et al*. 15 reported that CC chemokine receptor 4 (CCR4) is expressed on neoplastic cells of most patients with ATL and this expression is associated with cutaneous manifestation and poor prognosis. Recently, humanized anti‐CCR4 antibody (mogamulizumab) greatly improved ATL treatment in a phase II study 16. It takes a long time to develop new agents; however, several promising new agents, including an anti‐CCR4 antibody, are currently undergoing clinical trials associated with translational research. Furthermore, identifying alternative indications for known drugs is important for the pharmaceutical industry. As repositioning candidates have been through several phases of development for their original indication, drug repositioning offers the possibility of reduced time and risk; thus, several phases common to *de novo* drug discovery and development can be bypassed 17. This practice is highly attractive because of its potential to speed up the drug development process, thereby reducing costs and providing new treatments for unmet medical needs 18. With the successful clinical introduction of a number of noncancer drugs as cancer treatments, drug repositioning has become a powerful alternative strategy for discovery and development of novel anticancer drug candidates from within the existing drug space 19.

Peroxisome proliferator‐activated receptor‐γ (PPARγ) is a critical regulator of inflammation, adipocyte differentiation, glucose homeostasis, and tumorigenesis 20. PPARγ ligands have entered the clinical arena as therapeutic agents for epithelial and hematopoietic malignancies 21. Among clinically available angiotensin II receptor blockers (ARBs) commonly used to treat cardiovascular diseases, telmisartan is well known for its unique ability to activate PPARγ 22. Telmisartan inhibited cell growth of lung cancer cell lines via DNA‐binding activity of PPARγ, and induced annexin V‐positive apoptotic cells in urological cancer cell lines; however, the precise molecular mechanism of telmisartan‐induced cell death and the effect of telmisartan on primary cells remains unknown 23, 24. Here, we assessed how telmisartan affects ATL cells from patients and leukemia cell lines.

Here, we design to assess actions of telmisartan in primary ATL and AC cells, as well as leukemia cell lines. We found that telmisartan induced apoptotic cell death of primary ATL and AC cells, and leukemia cell lines. Telmisartan activated caspases and induced caspase‐independent cell death (CICD) by accumulation of LC3‐II, indicating autophagosome accumulation as well as autophagy type II cell death. A hypertension medication capable of exerting antiproliferation effects via apoptosis and autophagy in leukemia cells is intriguing. This is the first evidence demonstrating a cell growth‐inhibitory effect of telmisartan in fresh peripheral blood mononuclear cells (PBMCs) from leukemia patients.

## Materials and methods

### Clinical samples

Study subjects included two acute‐type ATL patients (median age 64 years, range 62–66, one male and one female), two chronic‐type ATL patients (median age 65 years, range 64–66, two females), one ATL patient in complete remission (CR; 79 years, female), three ACs (median age 64 years, range 52–77, one male and two females), and five healthy donors (HDs; median age 36 years, range 30–42, all males). ATL patients and ACs reported to the hospital for clinical examination of HTLV‐1 infections. Patients were examined by a standard serological testing for the presence of HTLV‐1 and by hematological/Southern blotting analysis for diagnosis of ATL. Those patients seropositive for HTLV‐1 without clinical symptoms of HTLV‐1‐related diseases were designated as ACs. Classification of ATL was made according to Shimoyama criteria 25. Clinical samples used in this study were acquired and handled in accordance with approved guidelines from the Committees for Ethical Review of Research involving Human Subjects at Kagoshima University. All patients gave their written informed consent for participation in this study and a review of their medical records, and provided a sample of peripheral blood for isolation of PBMCs. PBMCs were isolated from peripheral blood by separation using Ficoll/Hypaque (Pharmacia, Uppsala, Sweden) density gradient centrifugation at 400 ***g*** for 30 min. Fresh PBMCs were used for western blotting, reverse‐transcriptase PCR, and apoptosis analysis. Remaining PBMCs were cryopreserved in liquid nitrogen until examination, as previously described 26, 27. Phenotypic analysis using a PE‐conjugated murine anti‐CD25 monoclonal antibody (mAb) and a Cy7‐conjugated murine anti‐CD4 mAb (both purchased from Beckman Coulter, Brea, CA, USA) was also performed as previously reported 26, 27.

### Cell lines

S1T (derived from an ATL patient) 28, MT‐2 (derived from normal human leukocytes transformed by leukemic T cells of a patient with ATL) 29, Jurkat (HTLV‐1‐negative T‐cell line), and HL60 (an acute myeloid leukemia cell line) were cultured in RPMI‐1640 medium supplemented with the following reagents: 100 U·mL^−1^ penicillin, 0.1 mg·mL^−1^ streptomycin, 2 mm l‐glutamine, and 10% heat‐inactivated fetal bovine serum.

### Reagents

Telmisartan, irbesartan, and valsartan were purchased from Tokyo Chemical Industry (Tokyo, Japan). We used caspase inhibitor Z‐VAD‐FMK [Medical and Biological Laboratories (MBL), Nagoya, Japan], STF‐62247 (Merck Millipore, Darmstadt, Germany), and Bafilomycin A1 (Adipogen, Epalinges, Switzerland).

Primary antibodies against apoptosis‐inducing factor (AIF), β‐actin, and histone H1 were purchased from Cell Signaling Technology (Beverly, CA, USA). Anti‐LC3 antibody was obtained from MBL. We used horseradish peroxidase‐conjugated secondary antibodies (Vector Laboratories, Burlingame, CA, USA).

### Apoptosis analysis

PBMC cell lines were treated with varying concentrations of reagents for various periods of time. Apoptotic cells were analyzed by staining with annexin V‐FITC (MBL) and 7‐amino‐actinomycin D (Beckman Coulter) by flow cytometry analysis using a Cell Analyzer EC800 (Sony, Tokyo, Japan) 30. To assay DNA fragmentation, we employed a MEBSTAIN^®^ Apoptosis TUNEL Kit Direct (MBL). Percentages of specific annexin V‐ or TUNEL‐positive cells were calculated as follows: % specific apoptotic cells = (annexin V‐ or TUNEL‐positive cells − spontaneous annexin V‐ or TUNEL‐positive cells)/(100 − spontaneous annexin V‐ or TUNEL‐positive cells) × 100.

### Detection of caspase activity

Pan‐caspase and caspase‐3, ‐8, and ‐9 activity was assessed using CaspTag^™^ Pan Caspase In Situ Assay Kit (EMD Millipore, Billerica, MA, USA), APOPCYTO Intracellular Caspase‐3, ‐8 Activity Detection Kit (MBL), and CaspGLOW^™^ Fluorescein Active Caspase‐9 Staining Kit (BioVision, Milpitas, CA, USA) according to the manufacturer's instructions. In brief, 2 × 10^5^ cells were cultured for 60 min in the presence of fluorescent‐labeled caspase inhibitor, then subsequently washed and analyzed by flow cytometry 31.

### Cell viability assay

We assessed the effects of telmisartan on cell viability using water‐soluble tetrazolium (WST)‐8 (Wako Chemicals, Osaka, Japan), a cell proliferation reagent 32. Briefly, aliquots of 1 × 10^5^ cells·mL^−1^ (cell lines) or 1 × 10^6^ cells·mL^−1^ (PBMCs) were incubated in 96‐well plates in the absence or presence of telmisartan. After cultivation, we added 10 μL of WST‐8 for 2 h and measured absorbance at 450 nm (*A*_450_) using an Infinite^®^ 200 PRO (Tecan, Männedorf, Switzerland). Measurement of mitochondrial dehydrogenase cleavage of WST‐8 to formazan dye provided indication of relative cell viability levels.

### Mitochondrial transmembrane potential assay

We assessed mitochondrial transmembrane potential (Δψm) using a JC‐1 Mitochondrial Membrane Potential Assay Kit (Cayman Chemical Co., Ann Arbor, MI, USA). Briefly, cultured cells were incubated with JC‐1 staining solution for 30 min. Functional mitochondria containing red JC‐1 J‐aggregates, as well as apoptotic cells were analyzed according to the manufacturer's recommended method 31.

### Reactive oxygen species detection

Measurement of reactive oxygen species (ROS) was performed using the carboxy derivative of fluorescein, carboxy‐H_2_DCFDA (C‐400), which carries additional negative charges that improve its retention compared with noncarboxylated forms. Cultured cells were incubated with prewarmed PBS containing the probe (final dye concentration = 10 mm) for 30 min. Cell suspensions were analyzed using a Cell Analyzer EC800 31.

### Cell cycle analysis

Cell cycle analysis was performed with a DAPI Prep‐DNA staining solution (Sony). Briefly, cells were washed with PBS and incubated with DAPI Prep‐DNA staining solution for 30 min at room temperature. Cell suspensions were analyzed using a Cell Analyzer EC800 30.

### Protein extraction and western blotting analysis

We obtained whole‐cell extracts using RIPA Lysis Buffer (Santa Cruz Biotechnology, Santa Cruz, CA, USA) and were used immediately or stored at −80 °C. Cytoplasmic and nuclear extracts were obtained using NE‐PER Nuclear and Cytoplasmic Extraction Reagents (Pierce Biotechnology, Rockford, IL, USA) according to the manufacturer's protocols. Cell extracts were subjected to SDS/PAGE, electroblotted onto Immobilon^®^‐P membranes (EMD Millipore) and analyzed for immunoreactivity with appropriate primary and secondary antibodies, as indicated in figures, using Can Get Signal^®^ Solution (Toyobo, Osaka, Japan). Reaction products were visualized using Chemi‐Lumi One L (Nakalai Tesque, Kyoto, Japan) according to the manufacturer's protocols 30.

### Autophagy analysis by flow cytometry

We assessed autophagy using a Cyto‐ID Autophagy Detection Kit (Enzo Life Sciences, Farmingdale, NY, USA) according to the manufacturer's instructions 33, 34. Briefly, the 488‐nm excitable Cyto‐ID green autophagy detection reagent becomes brightly fluorescent in vesicles produced during autophagy, serving as a convenient tool for detecting autophagy at the cellular level. Autophagy analysis was performed by incubating cells with the autophagy flux inhibitor for 30 min at 37 °C, washing cells, and analyzing fluorescence by flow cytometry 31.

### Statistical analysis

Data are expressed as mean ± standard deviation (SD). For data analysis, Student's *t*‐test, Mann–Whitney *U*‐test, and Wilcoxon matched pairs tests were performed using Excel 2007 (Microsoft Japan, Tokyo, Japan) and statcel2 software (OMS Publishing, Tokyo, Japan). In all tests, *P* < 0.05 was considered statistically significant.

## Results

### Telmisartan induces cell death in primary ATL cells

**Figure 1:**
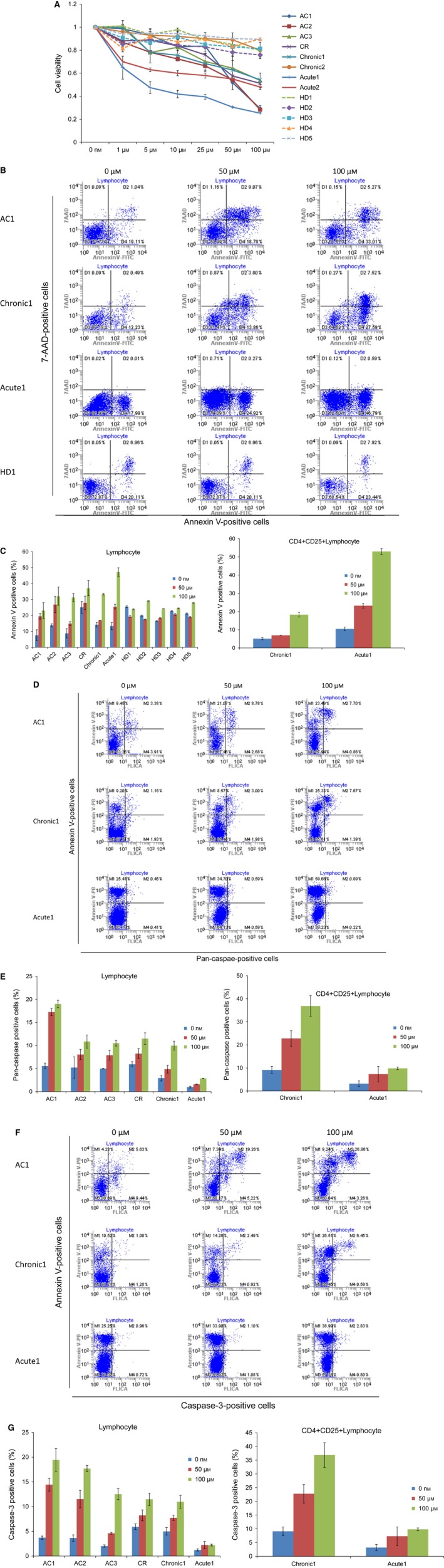
Telmisartan reduces cell viability and induces annexin V‐positive cells in PBMCs. (A) PBMCs were incubated at 1 × 10^6^ cells·mL^−1^ in the presence of indicated concentrations of telmisartan for 96 h (acute ATL,* n* = 2; chronic ATL,* n* = 2; CR,* n* = 1; AC,* n* = 3; HD,* n* = 5). Viability of cultured cells was measured by WST‐8 assay. (B–G) Cells cultured in the absence of telmisartan were assigned a relative viability of 1. Annexin V‐, 7‐amino‐actinomycin D‐ (7‐AAD), pan‐caspase‐, and caspase‐3‐positive cells were detected by flow cytometry in lymphocytes cultured for 72 h. Data represent mean ± SD of three independent experiments.

To investigate effects of telmisartan on primary ATL cells, we examined whether telmisartan affects viability of PBMCs from all subjects (two acute‐type ATL patients, two chronic‐type ATL patients, one ATL patient in CR, three ACs, and five HDs). Fresh PBMCs from acute ATL patients were more sensitive to telmisartan, whereas PBMCs from HDs were not sensitive (Fig. 1A). Telmisartan showed effective activities with average GI_50_ values of 51.6, 55.6, 3.8, and 82.0 μm against PBMCs from AC1, AC2, Acute1 and Acute2 patients, respectively.

To assess whether inhibition of cell growth occurred through enhanced apoptosis, PBMCs from subjects (one acute‐type ATL patient, one chronic‐type ATL patient, one ATL patient in CR, three ACs, and five HDs) treated with 50‐ or 100‐μm telmisartan were stained with annexin V (Fig. 1B,C). Percentages of specific annexin V‐positive cells in lymphocytes after treatment with 100‐μm telmisartan were 16.8% (AC1), 21.2% (AC2), 24.7% (AC3), 16.0% (CR), 22.4% (Chronic1), 40.2% (Acute1), and 5.8% (average of HDs). ATL cells possessed the function of regulatory T cells (Tregs) and expressed a CD4+/CD25+/FOXP3+/CCR4+ phenotype similar to Tregs 35. Percentages of specific apoptotic cells in CD4+/CD25+ lymphocytes after treatment with 100‐μm telmisartan were 14.9% (Chronic1) and 47.5% (Acute1). Telmisartan treatment resulted in a significant increase in the number of apoptotic primary acute ATL cells; the most sensitive was Acute1 with 85.0% ATL cells, while Chronic1 had 43.0%.

We also assessed effects of telmisartan on pan‐caspase (Fig. 1D,E) and caspase‐3 (Fig. 1F,G) in PBMCs from an acute‐type ATL patient, chronic‐type ATL patient, ATL patient in CR, and ACs. Fluorochrome‐labeled caspase inhibitors have consistently been shown to be highly reliable reporters of caspase activation and convenient markers of apoptotic cells 36. Telmisartan activated pan‐caspase and caspase‐3 activity in PBMCs from ACs, chronic‐type ATL patient, and ATL patient in CR, while annexin V‐positive cells with CICD were detected in acute‐type ATL.

### Telmisartan induces cell death of HTLV‐1‐associated cell lines

**Figure 2:**
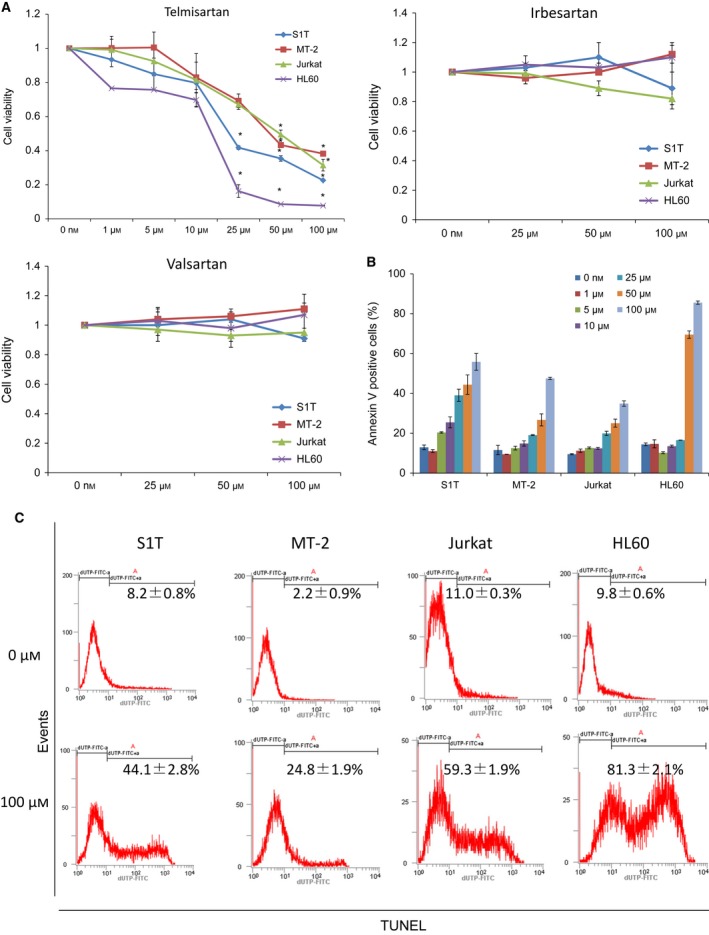
Telmisartan induces cell death of HTLV‐1‐associated cell lines. Cell lines were incubated at 2 × 10^5^ cells·mL^−1^ in the presence of indicated concentrations of ARBs for 72 h. (A) Proliferation of cell lines in the absence or presence of indicated concentrations of telmisartan, irbesartan, or valsartan were assessed by WST‐8 assay. Cells cultured in the absence of telmisartan were assigned a relative viability of 1. Data represent mean ± SD of three independent experiments. Apoptotic cells were detected as annexin V‐positive cells (B) and TUNEL‐positive cells (C) using flow cytometry. Data represent mean percentage of apoptotic cells ± SD of three independent experiments.

Next, we examined whether telmisartan affects cell viability of S1T, MT‐2, Jurkat and HL60 cells using a WST‐8 assay. Telmisartan inhibited growth of all four cell lines in a dose‐dependent manner, while ARBs, irbesartan, and valsartan did not inhibit cell growth (Fig. 2A). Telmisartan demonstrated potent activity with GI_50_ values of 20.1, 41.8, 48.1 and 13.9 μm for S1T, MT‐2, Jurkat, and HL60 cells, respectively.

We analyzed telmisartan‐induced cell death by annexin V (Fig. 2B) and TUNEL staining (Fig. 2C) to detect apoptosis. We observed that telmisartan induced annexin V‐positive cells in leukemia cell lines. Specific annexin V‐positive cells (%) after treatment with 100‐μm telmisartan were 49.3%, 40.6%, 28.1% and 83.1% for S1T, MT‐2, Jurkat and HL60 cells, respectively. Specific TUNEL‐positive cells (%) after treatment with 100‐μm telmisartan were 31.2%, 17.4%, 41.4% and 80.4% for S1T, MT‐2, Jurkat and HL60 cells, respectively.

### Telmisartan induces dysfunction of mitochondrial transmembrane potential and ROS generation

**Figure 3:**
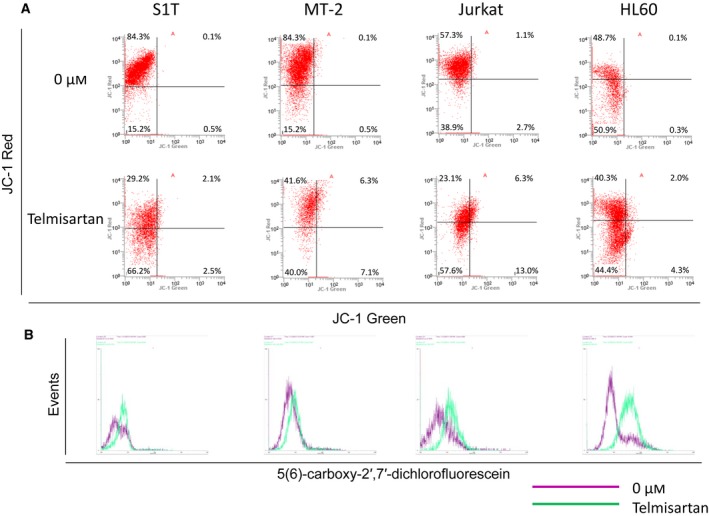
Telmisartan induces loss of mitochondrial transmembrane potential and ROS generation. S1T, MT‐2, Jurkat and HL60 cells were treated with telmisartan (100 μm) for 24 h. (A) Cells were analyzed for JC‐1 green and JC‐1 red fluorescence emission components by flow cytometry. (B) ROS levels were determined by assaying the fluorescent product, 5(6)‐carboxy‐2′,7′‐dichlorofluorescein, in viable cells by flow cytometry. Three independent experiments were performed per cell line, with representative results presented.

Release of caspase activators, such as cytochrome c, and loss of Δψm in mitochondria occur during apoptosis 37. JC‐1 spontaneously forms complexes showing intense red fluorescence in healthy cells with high Δψm. In contrast, JC‐1 remains in its monomeric form and displays green fluorescence in apoptotic or dead cells with low Δψm. Mitochondrial polarization was readily detected in telmisartan‐treated cells by measuring the shift in fluorescence emission by flow cytometry (Fig. 3A). Notably, telmisartan‐treated cells almost showed red fluorescence degradation, reflecting low Δψm.

Proteins released from the mitochondrial intermembrane space exert multifaceted effects, including caspase activation, chromatin condensation, DNA strand breakage, and ROS generation 38. We determined ROS levels by measuring oxidation of nonfluorescent carboxy‐H_2_DCFDA to the highly fluorescent 5(6)‐carboxy‐2′,7′‐dichlorofluorescein to clarify effects of telmisartan on intracellular redox status. Telmisartan stimulated ROS formation in leukemia cell lines, especially in S1T, Jurkat and HL60 (Fig. 3B). However, a ROS inhibitor, *N*‐acetyl‐l‐cysteine, only inhibited cell death in the MT‐2 leukemic cell line (data not shown).

### Telmisartan causes G_1_ phase cell cycle arrest

**Figure 4:**
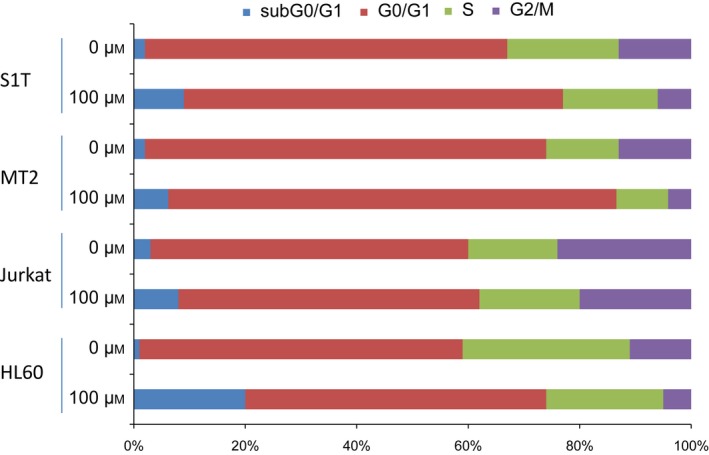
Telmisartan causes G_1_ phase cell cycle arrest. S1T, MT‐2, Jurkat, and HL60 cells were incubated in the absence or presence of telmisartan (100 μm) for 48 h, and then stained with DAPI and analyzed for DNA content by flow cytometry. Three independent experiments were performed per cell line; results are presented as mean percentages.

To further investigate the mechanism by which telmisartan inhibits growth of S1T, MT‐2, Jurkat and HL60 cells, we analyzed cell cycle distribution after exposing cells to telmisartan (Fig. 4). Cells were incubated with 100‐μm telmisartan for 48 h, and then subjected to cell cycle distribution analysis. Cultivation with telmisartan increased the population of cells in sub‐G_0_/G_1_ phase and markedly reduced cells in the G_2_/M phase, especially within S1T and HL60 cells. Apoptotic cells can be identified by DNA content frequency histograms as cells with fractional (sub‐G_1_) DNA content. Our results clearly show that telmisartan can induce apoptosis.

### Telmisartan induces both caspase‐dependent and caspase‐independent cell death

**Figure 5:**
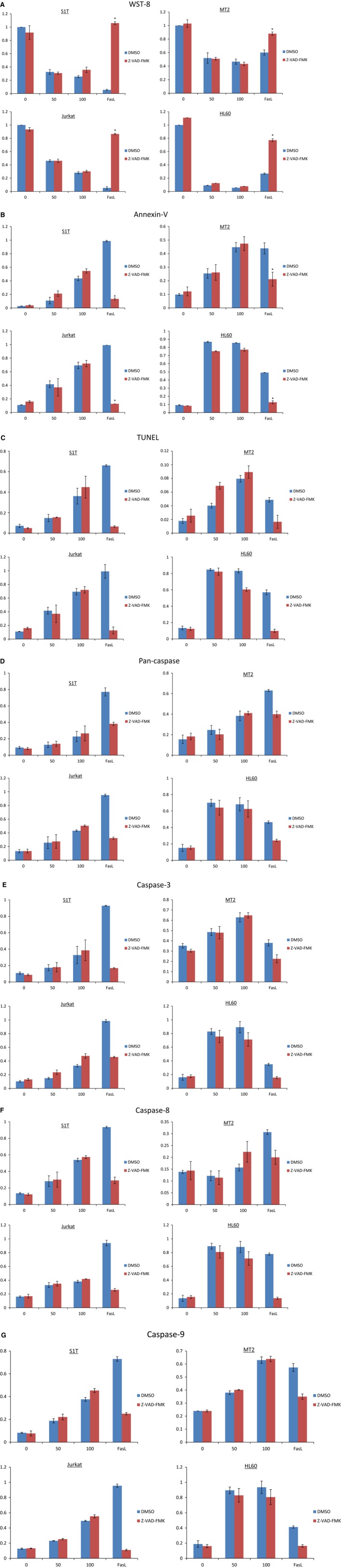
Telmisartan induces both caspase‐dependent and ‐independent cell death. (A–G) S1T, MT‐2, Jurkat and HL60 cells were treated with telmisartan (50 or 100 μm), anti‐Fas antibody (100 ng·mL^−1^), and Z‐VAD‐FMK (40 μm) for 72 h. Viability of cultured cells was measured by WST‐8 assay. (B–G) Annexin V‐, TUNEL‐, and caspase‐positive cells were detected by flow cytometry. Data represent mean percentage ± SD of three independent experiments. *P < 0.05 vs. each reagent in the absence of Z‐VAD‐FMK.

We analyzed the effects of a pan‐caspase inhibitor, Z‐VAD‐FMK, on telmisartan‐induced cell death (Fig. 5A). Telmisartan significantly inhibited cell growth and increased annexin V‐positive cells and DNA fragmentation in leukemia cell lines (Fig. 5B,C). Telmisartan also activated pan‐caspase, caspase‐3, ‐8 and ‐9 (Fig. 5D–G). While the pan‐caspase inhibitor did not inhibit cell death, annexin V‐positive cells, or DNA fragmentation, it did suppress Fas‐mediated cell death (Fig. 5A–C). Conversely, Z‐VAD‐FMK did not inhibit pan‐caspase or caspase‐3, ‐8 or ‐9 activities, but suppressed Fas‐mediated caspase activity (Fig. 5D–G). A calpain/cathepsin inhibitor was also incapable of inhibiting cell death in leukemic cell lines (data not shown).

### Telmisartan induces autophagy

**Figure 6:**
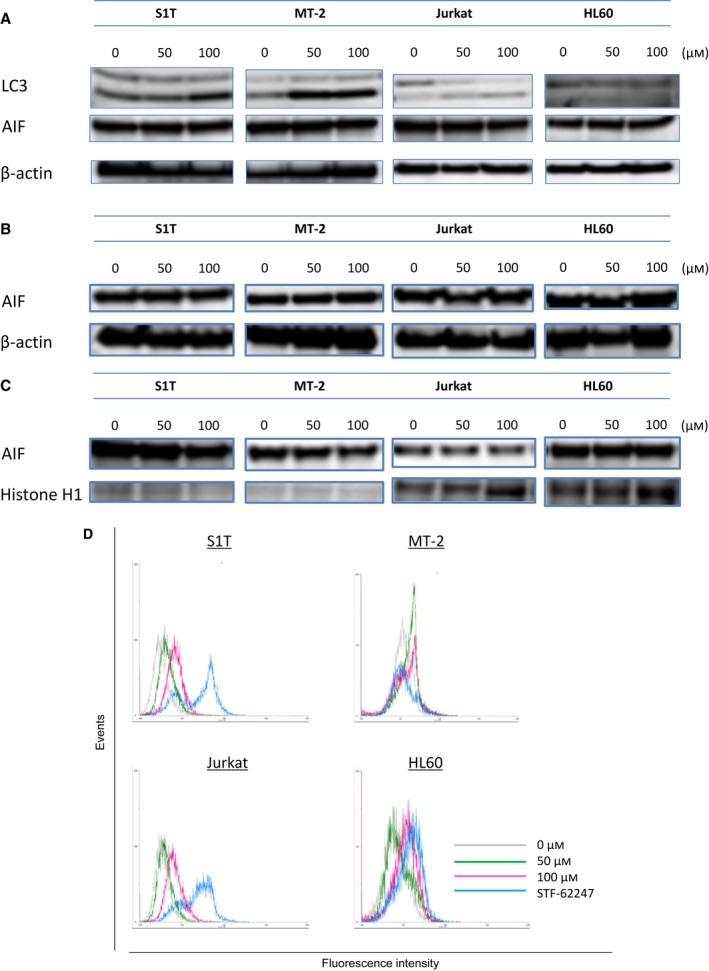
Telmisartan induces autophagy. (A–C) S1T, MT‐2, Jurkat and HL60 cells were treated with telmisartan (50 or 100 μm) for 48 h. Protein levels (A: whole cells; B: cytoplasmic; C: nuclear) were detected by western blotting with indicated antibodies. S1T, MT‐2, Jurkat and HL60 cells were treated with telmisartan (50 or 100 μm) for 72 h. (D) Cellular autophagic flux was evaluated using a Cyto‐ID autophagy detection kit. Cells were incubated for 30 min with the autophagy flux inhibitor provided.

Caspase inhibition does not maintain cellular viability, but instead shifts the morphology of death from apoptotic to nonapoptotic pathways such as autophagy and necrosis 39. Autophagy can catabolize cellular components, such that cells eventually activate apoptotic machinery. Conversion of the soluble form of LC3‐I to the autophagic vesicle‐associated form, LC3‐II, is considered to be a specific marker for autophagosome promotion. Telmisartan increased levels of LC3‐II (lipidated LC3) in leukemia cell lines (Fig. 6A).

In tumor cells with apoptotic defects, autophagy allows prolonged survival. Paradoxically, autophagy defects are associated with increased tumorigenesis; however, the mechanism behind this has not been determined 40. Multiple studies have shown that genetic knockdown of autophagy‐related gene (Atgs) or pharmacological inhibition of autophagy can effectively enhance tumor cell death induced by diverse anticancer drugs in preclinical models 41. In fact, telmisartan was synergized with 3‐methyadenine, an autophagy inhibitor, to induce cell death in leukemic cell lines after 48 h of culture (data not shown). Here, we assessed if autophagy occurs during telmisartan‐induced cell death. Autophagy levels increased in the presence of telmisartan or STF‐62247 when pretreated for 2 h with bafilomycin A1, which blocks fusion of autophagosomes with lysosomes (Fig. 6D).

Apoptosis‐inducing factor released as a result of mitochondrial outer membrane permeabilization (MOMP), which leads to release of proapoptotic proteins from the mitochondrial intermembrane space, can promote CICD through mechanisms that are poorly defined 42. AIF has been implicated in CICD following its translocation to the nucleus. AIF protein levels were stable in all telmisartan‐treated cell lines, as were cytosolic and nuclear AIF protein levels (Fig. 6A–C).

## Discussion

Despite recent advances in ATL treatments, monitored waiting until the disease progresses has been recommended for indolent ATL 9. Long‐term prognosis for this disease was inferior to that of, for example, chronic lymphocytic leukemia. Although relatively small, initial phase II studies and recent retrospective meta‐analyses have suggested that interferon‐α (IFN)/zidovudine (AZT) therapy may be promising for cancer types with leukemic manifestation, however, the therapeutic effects of IFN/AZT are not considered to be attributable to direct cytotoxic effects on leukemia cells 43, 44. Some patients with indolent ATL develop infections during the monitored wait. Prognosis for patients with aggressive ATL is one of the poorest among hematological malignancies, with overall survival at 3 years being only 24% for more aggressive ATL subtypes. Therefore, an urgent need exists for therapy and prophylaxis of conversion from AC to ATL, or from indolent to aggressive ATL. In our study, 1‐μm telmisartan induced 30–40% cell death in *ex vivo* PBMCs harvested from acute ATL patients (Fig. 1A). Clinically, the maximum concentration for oral administration of telmisartan (80 mg·day^−1^, 14 days) is 436.6 ng·mL^−1^ (0.8 μm). Therefore, oral administration of telmisartan may prevent progression from AC to ATL or from indolent ATL to aggressive ATL.

Interestingly, cell viability with 100‐μm telmisartan in AC1 (0% abnormal lymphocytes), AC2 (0.7% abnormal lymphocytes), and Acute1 (98.5% abnormal lymphocytes) fell below 40%. Furthermore, annexin V‐positive cells increased with pan‐caspase or caspase‐3 in AC1, but without these factors in Acute1 (Fig. 1B,D,F). Treatment with telmisartan slightly increased caspase activation in Chronic1 (43% abnormal lymphocytes). Thus, mechanisms of cell death may vary between AC and ATL patients. These results suggest that telmisartan induced caspase‐dependent cell death in HTLV‐1‐infected cells and CICD in ATL cells. Supporting this notion, a HTLV‐1 transactivator protein, HTLV‐1 Tax, has only been detected in < 40% of transformed ATL cells 45. Furthermore, caspase‐dependent apoptosis is inhibited at a CD95 death receptor proximal level in Tax‐expressing cells 46. Tax activates AKT in HTLV‐1‐transformed cells through the PI3 kinase pathway 47; the AKT pathway is also involved in targeting multiple proteins responsible for regulating caspase‐ and p53‐dependent apoptosis in HTLV‐1‐transformed cells. Kataoka *et al*. 48 reported the data of an integrated genomic and transcriptome analyses in an impressive cohort of 426 ATL cases. The elucidated alterations overlap significantly with the Tax interactome affecting T‐cell receptor and NF‐κB pathways. As such, mechanisms of cell death may also vary between cells with and without Tax. Although we have no data showing Tax expression in PBMCs and precise mechanisms remain unknown, telmisartan may interact with Tax protein regulation. Therefore, Tax‐expressing cells may be sensitive to caspase‐dependent cell death while acute‐type ATL patients have poor caspase activity.

Different types of cell death are often defined by morphological criteria, such as apoptosis, or simultaneous occurrence of autophagic vacuolization, necrosis, cornification, and tentative definitions of atypical cell death modalities 49. CICD can also occur despite efficient inhibition of caspases and can exhibit some morphological signs of apoptosis (such as partial chromatin condensation), autophagy, or necrosis. CICD correlates with loss of Δψm and reduction of ATP levels that precede mitochondrial release of AIF, suggesting MOMP donates to CICD primarily through loss of mitochondrial function 42. Following MOMP, Δψm is dissipated in both caspase‐dependent and ‐independent manners. Here, telmisartan treatment induced pan‐caspase, caspase‐3, ‐8 and ‐9 activities, and also reduced Δψm. Interestingly, the pan‐caspase inhibitor, Z‐VAD‐FMK, did not inhibit caspase‐dependent cell death and DNA fragmentation, indicating telmisartan simultaneously induced caspase‐dependent and ‐independent cell death mechanisms in leukemia cell lines. These results suggest molecules involved in CICD may augment caspase activity, such as secondary caspase activation. Thus, CICD induces caspase activation; however, the precise mechanisms have not yet been elucidated.

Autophagy is characterized by sequestration of cytoplasmic material within autophagosomes for bulk degradation by lysosomes 50. Autophagic cell death is mainly a morphologic definition (i.e., cell death associated with autophagosomes/autolysosomes), and there is still no conclusive autophagy pathway identified a cause of nonapoptotic cellular demise 40. Tumor cells with defects in autophagy demonstrate prolonged survival. Paradoxically, autophagy defects are associated with increased tumor development; however, the underlying mechanisms have not been determined. Presumed to result from excessive levels of cellular autophagy, ‘autophagic cell death’ is defined as a type of cell death occurring in the absence of chromatin condensation, described as a form of programmed cell death morphologically distinct from apoptosis. MOMP initiates removal of permeabilized mitochondria by autophagic machinery 42. While caspase inhibition does not completely prevent cell death, it often leads to a shift in the morphology of cell death, from appearance of classical apoptosis to apoptosis‐like cell death, autophagic cell death, or even necrosis 39. We assessed the possibility that telmisartan‐induced cell death occurs through autophagic mechanisms. Telmisartan increased LC3‐II levels and autophagic flux, and decreased loss of Δψm and caspase activation. Multiple studies have shown that genetic knockdown of Atgs or pharmacological inhibition of autophagy can effectively enhance tumor cell death induced by diverse anticancer drugs in preclinical models 41. In fact, telmisartan was synergized with 3‐methyladenine, an autophagy inhibitor, to induce cell death in leukemic cell lines within 48 h of culture (data not shown). These results indicate that telmisartan effectively induce cell death, causing caspase activation and autophagic flux; although, it is not yet known whether caspase activation requires autophagic type II cell death.

MT‐2 cells were most resistant to telmisartan‐induced cell death (Fig. 5) as MT‐2 is the most efficient at accumulating LC3 forms. MT‐2 is an HTLV‐1 Tax‐producing HTLV‐1‐infected T‐cell line derived from normal human leukocytes and transformed by leukemic T cells from a patient with ATL. HTLV‐1 Tax activates NF‐κB by stimulating IKK complex activity, a key regulator of NF‐κB signaling 12. Ren, *et al*. 51 showed that Tax‐deregulated autophagy is crucial for survival and proliferation of HTLV‐1‐transformed T cells. These results indicate HTLV‐1 Tax deregulates autophagy, although precise mechanisms have yet to be elucidated.

As a new strategy for drug discovery and development, drug repositioning offers the possibility of reduced time and risk, as several stages common to novel drug discovery can be bypassed. As safety and pharmacokinetic effects of existing medicines in humans have already been confirmed, these promising studies could provide safe and economic therapeutic agents to patients with speed and certainty. A hypertension medication capable of exerting antiproliferative effects on leukemia cells is intriguing. Our results suggest telmisartan is highly effective against HTLV‐1‐infected cells and ATL cells in a caspase‐dependent and ‐independent manner, respectively. Clinical use of telmisartan may suppress progression from indolent ATL or carrier status to aggressive ATL, thus improving the prognosis of patients with this fatal disease.

## Author contributions

TK designed and performed experiments, analyzed data, and wrote the manuscript. SS, AK, and NT also performed experiments and analyzed data. SH advised on experiments. HT, OI, SH, and SS supervised the project and contributed to manuscript development. MY and NA supervised the project and provided clinical samples.
